# Does acute promyelocytic leukemia patient with the STAT5B/RARa fusion gene respond well to decitabine? A case report and literature review

**DOI:** 10.1097/MD.0000000000022923

**Published:** 2020-10-23

**Authors:** Lei Wang, Xiaojing Yan, Juan He

**Affiliations:** Department of Hematology, The First Affiliated Hospital of China Medical University, Shenyang, Liaoning Province, China.

**Keywords:** acute promyelocytic leukemia, all-trans retinoic acid, PML-RARA, prognosis, STAT5B/RARa

## Abstract

**Rationale:**

Most acute promyelocytic leukemia (APL) patients respond to all-trans-retinoic acid (ATRA)and have a good prognosis. However, variants APL who carry PLZF/RARа, STAT5B/RARа, and STAT3/RARа are insensitive to ATRA and have poor prognoses. The standard treatment for variants APL is still unclear due to the small sample size.

**Patient concerns:**

Here we reported a Chinese male who was admitted to our hospital with the complaint of rib pain, dyspnea, and fever (37.5°C). Blood tests showed leukopenia (1.83 × 10^9^/L), anemia (hemoglobin 73 g/L), and thrombocytopenia (54 × 10^9^/L). Prothrombin time and activated partial thromboplastin time were normal.

**Diagnoses:**

The patient was diagnosed as STAT5b-RARa-positive APL based on the clinical and laboratory findings.

**Interventions:**

ATRA was used immediately for induction treatment, then he was treated with ATRA + arsenic trioxide and got the severe cardiac insufficiency. Subsequently, consolidation chemotherapy was added with ATRA + Huangdai tablets + idarubicin and decitabine, cytarabine, aclamycin (DCAG).

**Outcomes:**

The patient relapsed soon after his first molecular complete remission (CRm), fortunately, he got a second CRm with DCAG. He has survived for more than 9 months and remains CRm, now he is looking for a suitable donor to prepare for hematopoietic stem cell transplantation (HSCT).

**Lessons:**

APL patients with STAT5B-RARa is not only resistant to ATRA, but also to conventional combination chemotherapy such as daunorubicin and cytarabine/idarubicin and cytarabine or other regimens. Relapse and extramedullary infiltration is common, HSCT is a effective treatment, and the best time for HSCT is after the first CR. It should be noted that this patient got CRm with DCAG after relapse, so the role of decitabine in APL with STAT5B-RARa needs to be considered.

## 1 Introduction

More than 98% of acute promyelocytic leukemia (APL) patients have classic t(15,17)(q22;q12-23) and promyelocytic leukemia-retinoic acid receptorА (PML-RARА) fusion genes; they respond to all-trans retinoic acid (ATRA) and have a good prognosis. However, RARа fuses with other partner genes in patients with variants APL (1%–2%), and some new fusion proteins have been formed, such as PLZF/RARа, NPM/RARа, NuMA/RARа, STAT5B/RARа, FIP1L1/RARa, PRKAR1A/RARa, and BCOR/RARa. APL patients with PLZF/RARа, STAT5B/RARа, and STAT3/RARа are insensitive to ATRA.^[1]^ Patients with variants APL often have poor prognoses. The STAT5B/RARа fusion gene is rare in APL and was first reported in 1996.^[2]^ To date, 16 cases have been reported. The standard treatment for variants APL is still unclear due to the small sample size. Here, we report a 62-year-old man diagnosed as APL with STAT5B/RARа and summarize the clinical features and effective treatment, hoping to improve the prognosis of these patients.

## 2 Case report

**Figure 1:**
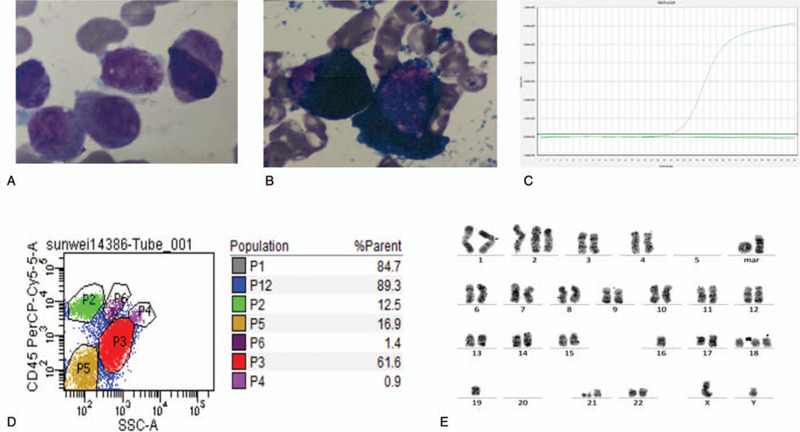
Laboratory examination result of this patient. (A) Abnormal promyelocyte in bone marrow (100× oil objective), (B) Peroxidase staining of bone marrow (100× oil objective), (C) Flow cytometric analysis of BM cells showing a population of abnormal cell (P3: 61.6%). (D) STAT5B-RARa fusion transcript detected by RT-PCR analysis in this patient. (E) Karyotype of this patient:43,−46,XY,+2,−5,+8,14p+,−16,17q−,17q+,+18,−19, 20, 21, +mar1, +mar2 [CP5]/46,XY[15]. BM = bone marrow, RT-PCR = reverse transcription–polymerase chain reaction.

A 62-year-old man was admitted to the hospital with the complaint of rib pain, dyspnea, and fever (37.5°C). Blood tests showed the following results: white blood cell count 1.83 × 10^9^/L, hemoglobin 73 g/L, platelet count 54 × 10^9^/L, prothrombin time 13.6 seconds, activated partial thromboplastin time 38.8 seconds, fibrinogen 5.8 g/L, D-dimer 2.4 μg/mL, fibrinogen degradation products 8.15 μg/mL. Abnormal promyelocyte in bone marrow aspiration showed 80.4% of blast, and flow cytometry indicated mainly positivity for CD33 and myeloperoxidase, partially positivity for CD117, CD9, CD11b, CD13, CD15 and CD64; negativity for CD7, CD19, CD10, cCD3, cCD79a, CD16, CD123, HLA-DR, CD14, CD56, CD3, CD4, CD8, CD2, and CD11c. Cytogenetics showed 43,−46,XY,+2,−5,+8,14p+,−16,17q−,17q+,+18,−19,−20,−21,+mar1,+mar2[CP5]/46,XY[15] (Fig. 1A). STAT5B/RARа fusion transcript was detected by reverse transcription–polymerase chain reaction. Given the clinical and laboratory findings, he was diagnosed as APL with STAT5B/RARа and treated with ATRA monotherapy for the first course, and no granulocyte differentiation was observed. However, his fibrinogen degradation product and D-dimer levels improved and he got a complete remission (CR) after the first course. Then he was treated with ATRA + arsenic trioxide (ATO) during the second course. But unfortunately, he was transferred to the intensive care unit because of severe cardiac insufficiency (BNPmax 1361 pg/mL) on the 14th day of the second course. However, STAT5B/RARа was still positive (17.96%) after the second course. He received 3 courses of ATRA + Huangdai tablets (ATO oral forms) + idarubicin in the following consolidation treatment and got molecular complete remission (CRm) after the fourth course, but STAT5B/RARа turned to be positive (0.6%) again after the fifth course. Fortunately, he got a second CRm with DCAG (decitabine [15 mg/m^2^, D1-5], cytarabine [10 mg/m^2^, q12, D3-9], aclamycin [8 mg/m^2^, D3-6], G-CSF [200 ug/m^2^, D0-1, till white blood cell count >20 × 10^9^/L]). This patient is now looking for a suitable donor to prepare for hematopoietic stem cell transplantation (HSCT).

## 3 Discussion

STAT5B is located on chromosome (Chr)17q21.1-21.2 and mainly distributed in the cytoplasm and nucleus. It regulates cell proliferation and induces leukemia through JAK-STAT signaling pathways. STAT5B plays a role in preventing further differentiation of hematopoietic cells together with the corepressor complex of deacetylase, this complex cannot be released by ATRA. This may explain the resistance of APL with STAT5B/RARa to ATRA.

The current case showed a complex karyotype and an unsatisfactory treatment process. He was responsive to ATRA and ATO at the beginning and achieved CRm after the fourth course; however, he relapsed quickly (molecular biology relapse). To date, a total of 16 cases of APL with STAT5B/RARа have been reported worldwide; this disease is extremely rare (Table 1 ). Only 3 patients were female, and more samples are needed to explain the sex-bias phenomenon. The age at onset was generally young; only 2 patients were over 60 years old. STAT5B/RARа is often accompanied by complex karyotypes (13 cases) and a high frequency of DIC (16 cases). ATRA can improve coagulation function in some patients (case 3, 9, 17), but granulocyte differentiation was observed in only 1 patient (case 15). Extramedullary infiltration in APL with PML/RARa is a rare event; even in patients without CR, 2 cases reported developed into extramedullary infiltration after CR (case 4 was CNS relapse, case 8 was testis relapse) (Table 1 ). Most patients received ATRA (monotherapy (n = 9) or combined with ATO/chemotherapy (n = 7)) in the first course of treatment (Table 1 ), but only 1 (ATRA monotherapy) and 3 patients (ATRA combined with ATO/chemotherapy) achieved CR after the first course. The CR rate is obviously lower in APL patients with STAT5B/RARа than in those with PML/RARа. After treatment with DA, IA (idarubicin and cytarabine), FLAG (fludarabine, cytarabine, and G-CSF), CAG (cytarabine, aclamycin, G-CSF), medium dose Ara-C, and DCAG, 12 patients achieved once CR at least in the whole course of treatment, 5 patients relapsed (case 2, 4, 8, 10, and 17), 5 patients died (case 2, 4, 5, 8, and 14) (Table 1 ). The CR rate and total mortality rate were 35.3% (6/17) and 47.1% (8/17) in APL with STAT5B-RARa, in contrast to 95% and 5% in APL with PML-RARa, respectively (Table 1 ). The main reasons of death were infections, progressive disease, cerebral hemorrhage or transplantation-related complications. Notably, 6 patients remained relapse-free until the report, 3 of them received combined chemotherapy (case 3, 13, 17), and another 3 received HSCT (case 6, 12, and 16). Among the 17 patients in Table 1 , 7 patients received HSCT, and 4 patients died (the longest OS was 53 months). Patients failed to get CR when HSCT after relapse (case 2, 8), the best time for HSCT is after CR1. Until now, the standard treatment for variants APL was still unclear due to the small sample size. Therefore, it is critical to improve the prognosis of patients with new treatment methods. It should be noted that 2 patients (case 13, 17) got CR with decitabine + AA/IA or DCAG after relapse, and the role of decitabine in APL with STAT5B-RARa needs to be considered.

**Table 1:**
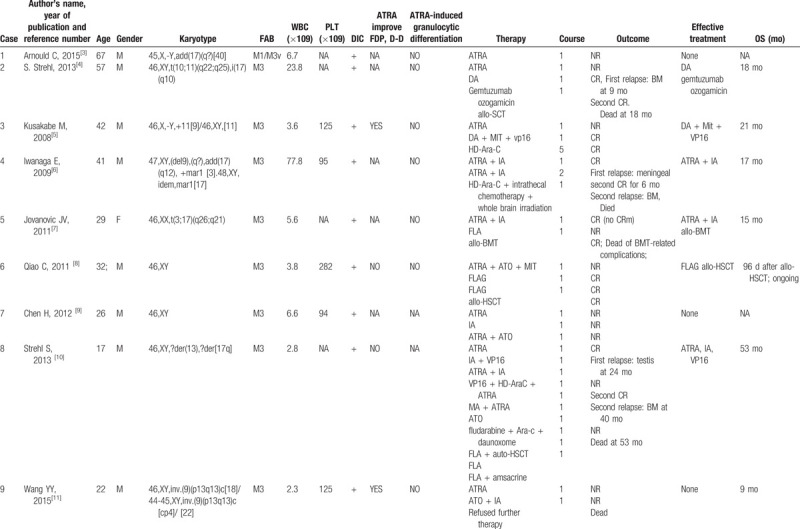
Clinical characteristics of APL patients with ATAT5B/RARa reported in the literature.

**Table 1 (Continued):**
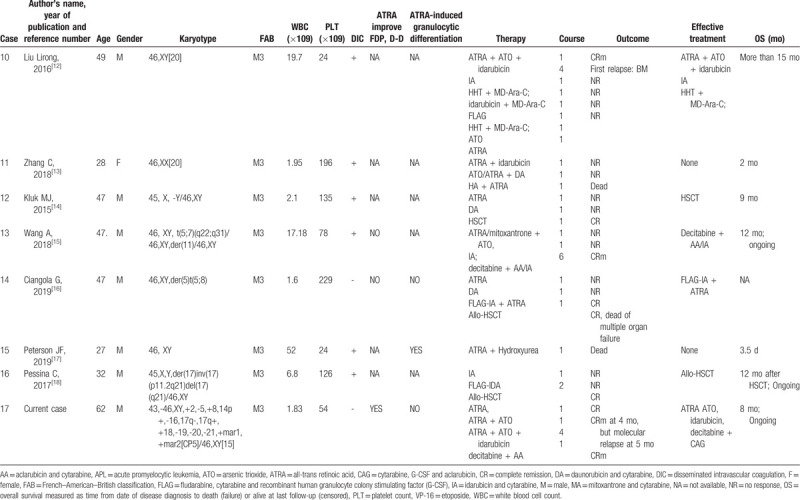
Clinical characteristics of APL patients with ATAT5B/RARa reported in the literature.

We can conclude that APL patients with STAT5B-RARa is not only resistant to ATRA, but also to conventional combination chemotherapy such as DA/IA or other regimens. Relapse and extramedullary infiltration is common, HSCT is a effective treatment, and the best time for HSCT is after the first CR.

## Acknowledgments

The authors would like to thank American Journal Experts who provided language editing.

## Author contributions

**Data curation:** Lei Wang.

**Funding acquisition:** Xiaojing Yan.

**Software:** Lei Wang.

**Writing – original draft:** Lei Wang.

**Writing – review & editing:** Lei Wang, Juan He.
